# Integrating GWAS and machine learning for disease risk prediction in the Taiwanese Hakka population

**DOI:** 10.3389/fgene.2025.1694084

**Published:** 2025-12-04

**Authors:** Jing-Hong Xiao, Hsiao-Yen Kang, Li-Ching Wu, Tien Hsu, Chin-Pyng Wu, Li-Jen Su

**Affiliations:** 1 Department of Biomedical Science and Engineering, National Central University, Taoyuan City, Taiwan; 2 Core Facilities for High Throughput Experimental Analysis, National Central University, Taoyuan City, Taiwan; 3 Department of Family Medicine, Department of Community Medicine, Landseed International Hospital, Taoyuan City, Taiwan; 4 Education and Research Center for Technology Assisted Substance Abuse Prevention and Management, National Central University, Taoyuan City, Taiwan; 5 Graduate Institute of Biomedical Sciences, China Medical University, Taoyuan City, Taiwan; 6 Critical Care Center, Department of Internal Medicine, Landseed International Hospital, Taoyuan City, Taiwan; 7 IHMed IVF Center, Taoyuan City, Taiwan

**Keywords:** type 2 diabetes, genome-wide association studies, machine learning, algorithmic rules, disease risk prediction

## Abstract

**Introduction:**

Genome-wide association studies (GWAS) have identified numerous loci associated with complex diseases, yet their predictive power in small or genetically homogeneous populations remains limited. Integrating machine learning with GWAS offers a path to improve risk prediction and uncover functional variants relevant to precision medicine.

**Methods:**

DNA samples from Taiwanese Hakka individuals with type 2 diabetes, hypertension, and eye diseases were analyzed. After standard quality control, 295,589 SNPs were retained. Fourteen machine-learning algorithms were evaluated using SNPs selected through traditional GWAS filtering and refined via wrapper-based feature selection with a best-first search algorithm. Model performance was assessed by internal cross-validation and external validation using Taiwan Biobank data, and functional annotation was conducted through GTEx v10 cis-eQTL analysis.

**Results:**

Predictive models relying solely on significant GWAS SNPs achieved moderate internal accuracy but limited generalizability. Incorporating feature-selected SNPs markedly improved performance: the Random Forest model achieved accuracies above 88% in cross-validation and above 85% in external validation, confirmed by 1,000× bootstrap resampling. eQTL analysis identified functional associations such as rs12121653-KDM5B and rs12121653-MGAT4EP, implicating pathways involved in metabolic and mitochondrial regulation.

**Discussion:**

These findings demonstrate that integrating GWAS with machine-learning-based feature selection enables the construction of robust, population-specific disease risk models. Given the small sample size of the discovery cohort (n = 96), all predictive results should be interpreted as exploratory. We employed stringent cross-validation and 1,000× bootstrap resampling to reduce overfitting, and genomic control metrics (QQ plots and λGC values) were evaluated to ensure no major test statistic inflation. Independent large-scale validation will still be required. The approach effectively captures additive and interaction-driven genetic components and provides a scalable framework for applying precision medicine to underrepresented or isolated populations.

## 1 Introduction

The global trend of population aging has led to a rapid increase in the prevalence of Non-communicable diseases (NCDs), contributing to the growing phenomenon of multimorbidity, whereby individuals concurrently suffer from multiple NCDs (Forouzanfar, 2016; Zhao et al., 2020). Currently, approximately one-third of the adult population worldwide is affected by multimorbidity, which not only places substantial strain on healthcare systems but also imposes significant economic burdens (Chowdhury et al., 2023; Hajat and Stein, 2018; Marengoni et al., 2011; Nguyen et al., 2019). This issue is particularly pronounced in low- and middle-income countries, which bear nearly 80% of the global NCD burden (Organization, 2013). Against this backdrop, elucidating the shared genetic underpinnings of diseases and developing precise predictive tools have emerged as critical priorities in precision medicine, with special relevance for populations possessing unique genetic backgrounds. Among NCDs, the global rise of diabetes has emerged as a significant public health crisis. According to the International Diabetes Federation (IDF) 2021 report, the prevalence of diabetes among individuals aged 20–79 worldwide has reached 10.5% and is projected to further increase to 12.2% by 2045 (Sun et al., 2022). Patients with diabetes are at elevated risk for a range of severe complications, including cardiovascular disease, kidney disease, eye disorders, neurological damage, lower-limb amputations, pregnancy-related complications, and premature mortality (Flores-Le Roux et al., 2011; Plantinga et al., 2010; Spijkerman et al., 2003; Organization, 2009).

GWASs have played crucial roles in elucidating the genetic mechanisms underlying diseases and in identifying potential therapeutic targets (Sachidanandam et al., 2001). Research has demonstrated that the development of Type 2 diabetes mellitus (T2D) and conditions such as obesity are influenced by the combined effects of multiple single-nucleotide polymorphisms (SNPs). However, many of these SNPs are located in noncoding regions of the genome and may indirectly contribute to disease development through the regulation of tissue-specific gene activity (Visscher et al., 2017; Schierding et al., 2018). Despite these findings, the functional mechanisms of SNPs in non coding regions remain largely unclear. Furthermore, most existing disease risk prediction models rely heavily on non-genetic factors, such as lifestyle and family history, which limits their predictive accuracy (Jostins and Barrett, 2011; Wang et al., 2016). Traditional GWAS approaches focus primarily on detecting the marginal effects of individual variants and are limited in their capacity to capture potential interactions among SNPs. Therefore, there is an urgent need to develop more precise disease prediction models. The integration of machine learning techniques for multivariate analysis and feature selection has been recognized as a promising strategy to increase predictive accuracy. Despite advances in genome-wide association studies (GWAS) and polygenic risk scoring, predictive models for small and genetically isolated populations remain underdeveloped. Traditional GWAS approaches often fail to generalize due to limited statistical power and the inability to capture complex SNP interactions. To address this limitation, we integrated GWAS with machine learning-based feature selection to construct robust, population-specific disease risk models in the Hakka population of Taiwan.

The Hakka population originates in northern China, with significant migration to Taiwan occurring in the 17th century. The Hakka community has traditionally practiced endogamy, leading to the formation of a population with a distinctive genetic background. According to statistics from the Hakka Affairs Council of Taiwan in 2021, the Hakka population in Taiwan exceeds 4.66 million, accounting for approximately 19.8% of the total Taiwanese population, and is primarily concentrated in regions such as Taoyuan, Hsinchu, and Miaoli. The relative genetic isolation of Hakka has contributed to a higher prevalence of certain genetic disorders, including hypertension and thalassemia (Cai et al., 2023; Wu et al., 2022; Lin et al., 2013; Chien et al., 1992), and has been shown to influence their response to statin therapy (Zhong et al., 2018). Given the high genetic homogeneity and unique disease prevalence within the Hakka population, they represent an ideal group for investigating associations between genetic variations and disease risk, making them particularly well-suited for studies in precision medicine. Although GWASs have demonstrated significant success in large populations with high genetic diversity, considerable challenges remain when GWASs are applied to studies involving small sample sizes or relatively isolated populations. For example, isolated populations such as those in the Faroe Islands exhibit high levels of genetic homogeneity due to consanguinity, which further complicates GWAS analyses (Gislason, 2023). To address these challenges, recent studies have explored the integration of protein-protein interaction networks and gene expression data to improve predictive accuracy in the context of limited sample sizes (Guo et al., 2021). Additionally, optimizing the ratio of cases to controls has been shown to increase the robustness of statistical analyses (Hong and Park, 2012). Genetic risk prediction models are commonly developed using polygenic risk scores or machine learning approaches (Wei et al., 2009; Abraham and Inouye, 2015). The predictive performance of these models is typically evaluated via receiver operating characteristic (ROC) curves (Jostins and Barrett, 2011; Wang et al., 2016; Vihinen, 2013; Kooperberg et al., 2010). By identifying and selecting the most informative features from genotype data, it is possible to achieve optimal prediction accuracy for the target disease (Quinlan, 1990; Schapire and Freund, 2012; Okser et al., 2014). Advanced models often employ gradient descent and iterative parameter optimization procedures to enhance predictive performance, continually refining the model until convergence on an optimal solution is achieved (Yuan, 2008; Mehta et al., 2019). Ultimately, predictive models are subject to validation to determine their effectiveness in disease risk prediction (Abraham and Inouye, 2015; Vihinen, 2012). However, genome-wide analyses in genetically homogeneous groups often suffer from low statistical power due to limited sample size. These constraints necessitate careful modeling strategies and rigorous validation to avoid overfitting and to ensure reproducibility. In this context, the present study should be viewed as a methodological pilot rather than a definitive association-mapping effort.

This study focused on the Hakka population in Taiwan, utilizing data from Pingzhen District Hospital and the Taiwan Biobank (TWB). By integrating innovative SNP selection strategies with advanced machine learning techniques, this study aimed to overcome the inherent challenges associated with small-sample research. In predicting the risk of T2D, hypertension, and eye diseases, the random forest model demonstrated particularly strong performance, achieving high predictive accuracy in both cross-validation and external validation analyses. These findings not only deepen our understanding of the unique genetic background of the Hakka population but also underscore the importance of incorporating population-specific genetic information into precision medicine frameworks, thereby laying a critical foundation for future clinical applications and personalized healthcare. In summary, this study successfully combined GWAS and machine learning methodologies, with a focus on the Hakka population as the core study group. This research established a population-adapted disease risk prediction model and revealed potential genetic mechanisms underlying disease susceptibility, providing a robust scientific basis for the advancement of precision medicine.

## 2 Materials and methods

### 2.1 Study population and data preprocessing

**FIGURE 1:**
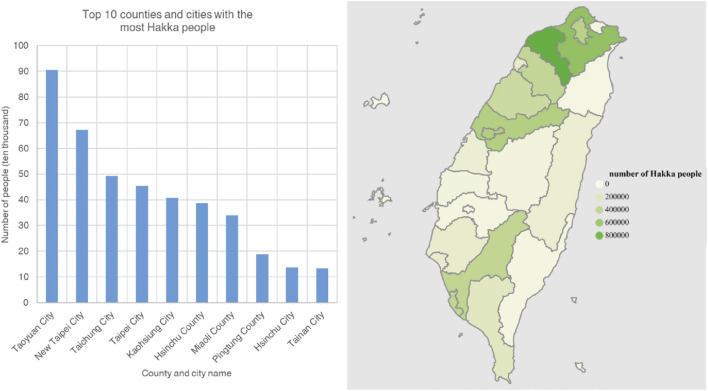
Distribution Map of the Hakka population in the Taiwan Region. This image illustrates the distribution of the Hakka population across various counties and cities in Taiwan. The bar chart on the left shows the top 10 counties and cities with the largest Hakka populations. On the right, a map visually represents the distribution of the Hakka population across different regions of Taiwan. Different shades of color indicate varying numbers of Hakka people; darker color represent higher Hakka populations. The map reveals that the Hakka population is primarily concentrated in the northern and central regions, with Taoyuan city, New Taipei city, and Taichung city being the main areas of settlement.

The training dataset for this study was derived from the Landseed Integrated Outreaching Neighborhood Screening, (LIONS) project, which was initiated in 2005 by Landseed International Hospital in Pingzhen District, Taoyuan city, Taiwan (Chang et al., 2019). The primary aim of the LIONS project was to investigate chronic diseases and related risk factors among adults aged 30 years and older who had resided in the area for at least 30 years. Households were selected via probability sampling proportional to size. Taoyuan is a major settlement area for the Hakka population in Taiwan, and according to the 2021 Survey of Hakka Population and Language Use in Taiwan (Figure 1), approximately 905,000 Hakka individuals reside in this region. Therefore, this demographic context was crucial for the selection of our study samples. Data preprocessing encompassed outpatient and inpatient records collected by Landseed International Hospital between 2007 and 2015. The initial dataset comprised 4,426,698 clinical visits, representing 517,781 individuals. The preprocessing steps included: (1) removing records with inconsistencies in reported sex, and (2) excluding vulnerable populations such as children and pregnant women. After preprocessing, the final dataset consisted of 517,464 individuals, providing a solid foundation for subsequent analyses. For external validation, data from the TWB were utilized. The 96 Hakka individuals from the Landseed LIONS project constituted the sole discovery and training cohort for model development, and none of these individuals overlapped with participants from the Taiwan Biobank used exclusively for external validation. The TWB dataset includes extensive phenotypic information collected through a structured questionnaire system, encompassing demographic characteristics, socioeconomic status, environmental exposures, lifestyle behaviors, dietary habits, family medical history, and self-reported disease conditions. The detailed genotyping procedures have been previously described by Wei et al. (2021).

### 2.2 DNA sample preparation and genotyping

This study included DNA samples from 440 individuals who were diagnosed with hypertension, cataracts, or T2D, and represented multiple ethnic groups: Hakka (n = 242), Fujianese (n = 101), Hakka-Fujianese admixed individuals (n = 27), Indigenous Taiwanese (n = 8), and non-Taiwanese individuals (n = 62). To minimize potential genotyping errors arising from population heterogeneity, we focused subsequent analyses on the Hakka subgroup, which exhibited greater genetic homogeneity. DNA integrity was assessed via gel electrophoresis, and concentrations were standardized to 15–20 ng/μL via a NanoDrop 2000/2000c spectrophotometer. Supplementary Figure S1 summarizes the workflow and results. Among the 242 Hakka DNA samples, 84 were randomly selected from individuals diagnosed with the target diseases and related comorbidities for further analysis. In addition, 16 healthy Hakka individuals were selected as controls. The quality control (QC) procedures included confirming DNA integrity via electrophoresis and assessing potential contamination via spectrophotometric measurements, with acceptable optical density (OD_260/230_) ratios defined as >1.8 and 1.8 < OD_260/280_ < 2. As a result, four abnormal samples were excluded. Genotyping was performed via the Affymetrix C2-42 Axiom Genome-Wide TWB Array Plate, which generated raw genotypes for 752,921 SNPs, consistent with Supplementary Figure S2. Genotyping was conducted at the National Center for Genomic Medicine in Taiwan following the manufacturer’s protocols. Initial preprocessing included filtering on the basis of call rate, sex concordance, minor allele frequency, Hardy-Weinberg equilibrium, heterozygosity, and the exclusion of related individuals.

### 2.3 Genome-wide association studies (GWAS)

In the GWAS analysis of this study, we included genotype data from 96 Hakka DNA samples collected through the Pingzhen District Hospital population study (comprising 80 cases and 16 controls), as well as from 8,287 Hakka participants in the TWB. To ensure data quality and reliability, stringent quality control procedures were performed via PLINK (v1.9) (Purcell et al., 2007), following established protocols (Anderson et al., 2010; Marees et al., 2018). Standard GWAS quality control procedures were applied (detailed thresholds in Supplementary Figure S2), ensuring high-quality genotype data for downstream analyses. After quality control, 295,589 SNPs were retained from the hospital samples for subsequent analyses, ensuring the accuracy and robustness of the downstream analyses. Intermediate QC steps yielded 673,521 SNPs after missingness filtering and 295,624 SNPs after MAF filtering, before arriving at the final set of 295,589 SNPs used for GWAS. Supplementary Figure S2 summarizes the workflow and results. The GWAS findings were visualized via graphical representations such as Manhattan plots and Venn diagrams. In addition to reporting raw P-values, we indicated both Bonferroni-corrected and false discovery rate (FDR) significance thresholds in the Manhattan plots to account for multiple testing, although no single variant reached conventional genome-wide significance in this small cohort.

### 2.4 Covariate adjustment and genomic control

To address potential population structure and confounding, all primary GWAS models included sex as a covariate. In addition, we evaluated models incorporating the top principal components (PC1-PC3, PC1-PC5, and PC1-PC10). As shown in Supplementary Table S4, including additional PCs increased the genomic inflation factor (λGC), with values rising from approximately 1.02 (sex only) to as high as 1.15 (PC1-10). Given the very small discovery cohort and the relative genetic homogeneity of the Hakka population, this pattern likely reflects statistical instability from over-correction, rather than true population stratification. Therefore, following best-practice recommendations for small-sample GWAS, we retained the sex-adjusted model as the primary analysis. QQ plots and λGC values for all traits are presented in Figures 3d–f and Supplementary Table S4, demonstrating mild deviation from the null and no evidence of major test-statistic inflation.

### 2.5 Machine learning model development

This study evaluated fourteen widely used machine learning algorithms, including Naïve Bayes (John and Langley, 2013), LIBSVM (Chang and Lin, 2011), stochastic gradient descent support vector machine (SGD) (Platt, 1999), sequential minimal optimization for logistic regression (SMO) (Cessie and Houwelingen, 1992), k-nearest neighbors (KNN) (Aha et al., 1991), locally weighted learning (LWL) (Frank et al., 2012), RIPPER (Cohen, 1995), One Rule (Holte, 1993), PART (Frank and Witten, 1998), Zero Rule, C4.5 decision tree (Quinlan, 2014), logistic model trees (LMT) (Landw et al., 2005), Random Tree, and Random Forest (Breiman, 2001). To identify informative SNPs while minimizing redundancy, variants with P < 1 × 10^−2^ were first selected based on prior evidence suggesting that moderately significant SNPs may still contribute meaningful predictive signal (Narendra and Fukunaga, 1977; Montaez, 2018). Feature selection was then conducted using a wrapper-based procedure (Kohavi and John, 1997; John et al., 1994) combined with a best-first forward search strategy (Supplementary Figures S4 and S5). During this process, candidate SNP subsets were iteratively expanded, and each newly added SNP was evaluated according to its contribution to LOOCV accuracy. Variants that improved cross-validated performance were retained, and the search continued until no additional gain was observed. Importantly, this wrapper-based feature selection was performed once on the discovery cohort under LOOCV to obtain a stable and interpretable SNP subset for each disease. The resulting marker sets were then fixed for all subsequent analyses. For downstream robustness assessment, we focused on the best-performing classifier (Random Forest). Only the final Random Forest model was subjected to 10 × 10 repeated cross-validation (to evaluate internal stability) and to external evaluation through 1,000× stratified bootstrap resampling on the TWB Hakka cohort. Both procedures used the same fixed SNP subset and were performed without re-running feature selection, ensuring that robustness estimates reflected model stability rather than repeated optimization of the feature space. Feature selection and model tuning were performed exclusively in the discovery cohort; Taiwan Biobank samples were only used for external evaluation, ensuring a strict separation between training and validation data and preventing information leakage.

### 2.6 Expression quantitative trait loci (eQTL) analysis of disease-associated SNPs

To enhance the biological interpretation of disease-associated SNPs identified through GWAS, we cross-referenced significant variants with public data from the Genotype-Tissue Expression (GTEx) project, v10 (GRCh38). We retrieved its reported cis-eQTL associations directly from GTEx, defined within ±1 Mb of the gene transcription start site (TSS). We examined eQTL effects across all available tissues and summarized those most relevant to our phenotypes (e.g., adipose and skeletal muscle for T2D, arterial/whole blood for hypertension, and skin/ocular-related tissues for cataract/eye traits). Alleles and genomic coordinates were harmonized to the GRCh38 build following GTEx conventions (effect vs. non-effect allele). To avoid strand ambiguity, palindromic SNPs (A/T or C/G) with MAF ≥0.40 were excluded; non-palindromic sites were aligned by rsID and genomic position. All annotations are listed in Supplementary Table S3, including GTEx-derived fields such as gene_id, gene_name, tissue, slope, slope_se, pval_nominal, qval, and others. Only entries with q < 0.05 were considered significant and highlighted accordingly. The eQTL analysis was conducted as a *post hoc* biological interpretation to evaluate the functional relevance of SNPs identified by machine learning models, rather than being directly incorporated as features in the predictive modeling process.

## 3 Results

### 3.1 Comorbidity association analysis

**FIGURE 2:**
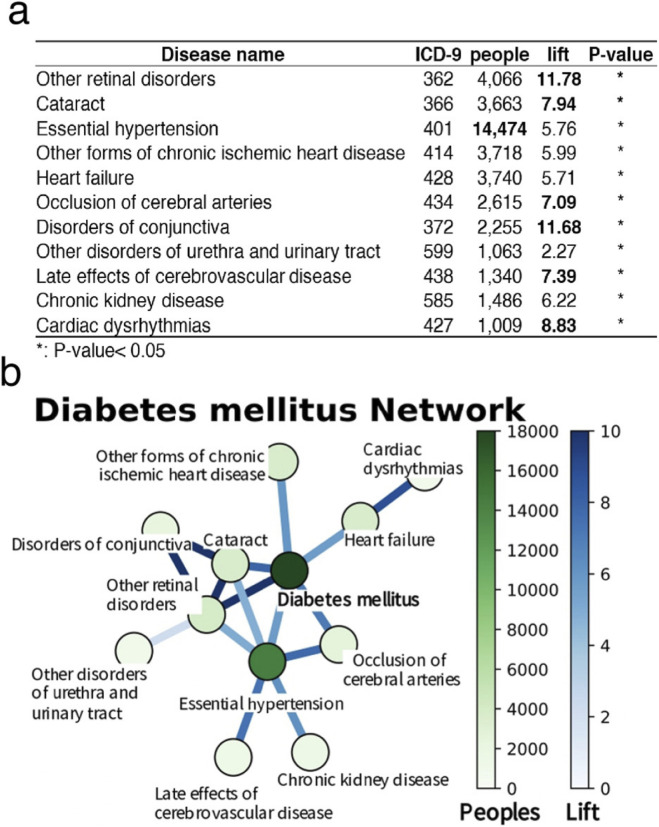
Results of association rule analysis for type 2 diabetes. **(a)** This table lists 11 diabetes-associated diseases, including the International Classification of Diseases (ICD-9) codes, the number of affected individuals, the lift, and the P-values for each disease. The lift indicates the ratio of the frequency of disease occurrence in diabetic patients to its frequency in the general population. A P-value less than 0.05 signifies statistical significance, with asterisks (*) in the table denoting diseases statistically significantly associated with diabetes. **(b)** The network diagram visually illustrates the relationships between diabetes and the diseases mentioned above. Each node in the diagram represents a disease, with the color and size of the node corresponding to the number of affected individuals. Darker color and larger nodes indicate a greater number of patients. The thickness of the edges represents the lift; thicker edges indicate a greater lift.

Association rule analysis was employed to investigate patterns of disease co-occurrence related to T2D within the Hakka population. The analysis revealed that hypertension was the most common comorbidity among individuals with T2D, with a prevalence rate of approximately 23% in outpatient records. Furthermore, eye diseases, including cataracts and diabetic retinopathy, presented the highest lift values, indicating that the risk of developing these conditions increased more than eightfold following the onset of diabetes. These associations were statistically validated using the chi-square test (P < 0.05). On the basis of these findings, T2D, hypertension, and eye diseases were selected as focal points for further analyses (Figure 2a). Network visualization (Figure 2b) further highlighted diabetes as a central condition, showing strong associations with common comorbidities such as hypertension, chronic kidney disease, and cardiovascular diseases, including heart failure and chronic ischemic heart disease. Although conditions such as cerebral artery occlusion and arrhythmia affect fewer individuals, their elevated lift values indicate abnormally high clustering within the diabetic population, warranting further investigation. These findings underscore the systemic nature of diabetes and emphasize the need for integrated disease management strategies, particularly those that target eye and cardiovascular complications.

### 3.2 GWAS findings

**FIGURE 3:**
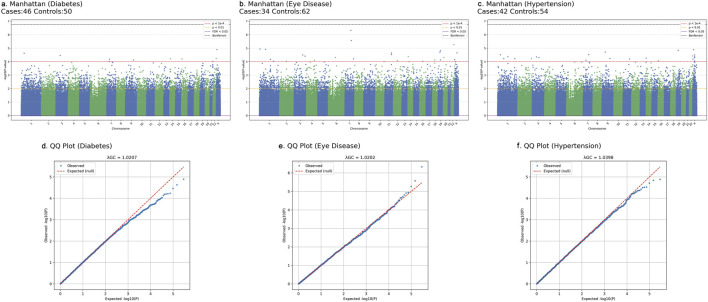
Manhattan and quantile-quantile (QQ) plots of GWAS results for three diseases in the Taiwanese Hakka discovery cohort. Association tests were performed using logistic regression adjusting for sex. **(a–c)** show Manhattan plots for **(a)** type 2 diabetes (46 cases, 50 controls), **(b)** eye disease (34 cases, 62 controls), and **(c)** hypertension (42 cases, 54 controls). Each point represents a single SNP plotted by genomic position (x-axis; chromosomes 1-22 and X) and -log_10_(P) value (y-axis), with adjacent chromosomes distinguished by alternating blue and green colors. Horizontal reference lines indicate commonly used significance thresholds: red dashed line, P = 1 × 10^−4^; orange dotted line, P = 0.01; purple dashed line, FDR <0.05; black dashed line, Bonferroni-corrected genome-wide significance. **(d–f)** show QQ plots for the corresponding traits, comparing observed versus expected -log_10_(P) values under the null (red dashed line). Genomic inflation factors (λGC) are reported at the top of each QQ panel (**(d)**: 1.0207 for diabetes; **(e)** 1.0202 for eye disease; **(f)** 1.0398 for hypertension), indicating minimal deviation from the null distribution and no evidence of major test-statistic inflation.

Following quality control, a total of 295,589 SNPs were retained for the GWAS analyses. For each trait (type 2 diabetes, hypertension, and eye diseases), we performed separate logistic regression GWAS and applied two exploratory significance thresholds. Using a stringent threshold (P < 1 × 10^−4^), the three trait-specific GWAS results yielded 10 (diabetes), 27 (eye diseases), and 20 (hypertension) associated SNPs. The union of these trait-specific sets contained 52 unique SNPs (Supplementary Figure S3). Using a more lenient threshold (P < 1 × 10^−2^), the GWAS yielded 2,848 (diabetes), 2,878 (eye diseases), and 2,883 (hypertension) associated SNPs, with a total of 5,973 unique SNPs in the combined union set (Supplementary Figure S3). These union sets served as the candidate feature pools for downstream machine-learning analyses, and the SNP selection results are summarized in Figure 3 and Supplementary Figure S3. Notably, we observed that several SNPs were shared across multiple diseases, suggesting the existence of common genetic mechanisms underlying these conditions. Across all three traits, the genomic inflation factors (λGC) from the sex-adjusted models ranged between 1.0202 and 1.0398, indicating minimal deviation from the null expectation. Models incorporating additional PCs showed progressively higher λGC values, reaching approximately 1.15, a pattern consistent with the known instability of PCA-based correction in small and genetically homogeneous samples. The QQ plots (Figures 3d–f) further support the adequacy of our GWAS calibration, showing near-null distributions with only mild tail inflation. These results suggest that the sex-adjusted model provides the most stable and interpretable association statistics for the present sample size. This phenomenon has been reported in other small-sample or isolated-population GWAS, where PCs may absorb genuine signal or introduce instability.

### 3.3 Initial model performance using GWAS-selected SNPs

The initial predictive models were constructed on the basis of 52 SNPs identified through GWAS. To increase model robustness and mitigate the risk of overfitting, we employed LOOCV to maximize the utilization of limited sample sizes. Additionally, data from the TWBs, which represent individuals from the same geographical region, were used to further assess the external predictive performance of the models in the Hakka population residing in Pingzhen, Taoyuan. The results indicated that the T2D model built via LIBSVM achieved high accuracy during internal cross-validation (91.67%). However, its performance decreased sharply to 53.85% when it was validated externally via the TWB dataset, suggesting overfitting and poor generalizability. In contrast, although the random forest model exhibited lower accuracy in internal validation (84.4%), it maintained higher accuracy in external validation (69.23%), demonstrating comparatively stronger robustness. For the prediction of eye diseases, both the LIBSVM and Zero Rule models demonstrated the highest performance in cross-validation, achieving an accuracy of 64.58%. However, during external validation, the C4.5 decision tree model yielded the highest accuracy, reaching 75%, despite its lower performance in internal validation (43.75%). Similarly, in the prediction models for hypertension, the KNN and One Rule algorithms achieved relatively high accuracy in internal cross-validation (62.5%). Nevertheless, in the external validation, the LWL model performed best, achieving an accuracy of 51.52% (Supplementary Table S1). These findings highlight the limitations of building predictive models on the basis solely of SNPs selected from GWASs, particularly when these models are applied to independent datasets, where performance often declines owing to insufficient generalizability.

### 3.4 Enhanced model performance through feature selection and machine learning integration

Recognizing the limitations of relying solely on highly significant SNPs, we initially attempted to construct models using all 295,589 SNPs. However, due to feature redundancy and computational constraints, internal performance improved only marginally and external validation yielded limited gains. To enhance predictive efficiency, we applied a more lenient significance threshold (P < 1 × 10^−2^) to include additional informative variants for model training. A wrapper-based feature selection strategy combined with a best-first search algorithm was implemented to iteratively identify compact yet informative SNP subsets (Supplementary Figures S4 and S5). This process aimed to reduce redundancy and improve model interpretability. Cross-validation was applied to mitigate overfitting, and external validation was conducted using the TWB Hakka cohort to assess generalizability. Across all evaluations, the Random Forest model consistently exhibited the best overall performance, achieving cross-validation accuracies above 88% and external validation accuracies above 85% (Table 1). The final feature sets were small but interpretable, including 14 SNPs for T2D, 10 for eye diseases, and 15 for hypertension (Supplementary Table S2). To compare model behavior under different feature-selection paradigms, we trained 14 classifiers using both GWAS-selected SNPs and machine-learning-based marker subsets. Feature selection was embedded within each training fold (leave-one-out) to prevent data leakage and ensure independent optimization for each model. This model-dependent process allowed each classifier to identify the subset of variants most informative for its decision boundaries, balancing predictive accuracy and biological interpretability.

**TABLE 1:** Presents the results of using machine learning to filter SNPs and establish models under different conditions, as well as the outcomes of model cross-validation and validation against TWB.

Model name	Accuracy (%)											
	Diabetes mellitus				Eye disease				Hypertension			
	Total SNP	GWAS selection	Best first	TWB	Total SNP	GWAS selection	Best first	TWB	Total SNP	GWAS selection	Best first	TWB
Decision tree (C4.5)	45.83	43.75	95.83	65.38	50	63.54	92.71	46.43	57.29	48.96	93.75	57.58
			(8 SNPs)				(12 SNPs)				(7 SNPs)	
KNN	46.88	100	97.92	61.54	54.17	100	97.92	35.71	46.88	100	100	60.61
			(7 SNPs)				(9 SNPs)				(9 SNPs)	
LIBSVM	52.08	100	95.83	65.38	64.58	100	100	53.57	56.25	100	96.88	66.67
			(10 SNPs)				(12 SNPs)				(9 SNPs)	
Logistic model trees	60.42	56.25	100	65.38	59.38	75	100	50	58.33	62.5	96.88	54.55
			(10 SNPs)				(11 SNPs)				(7 SNPs)	
LWL	69.79	62.5	89.58	42.31	57.29	82.29	87.5	53.57	75	60.42	94.79	39.39
			(10 NPs)				(5 SNPs)				(7 SNPs)	
NaiveBayes	40.63	100	100	65.38	64.58	100	98.96	60.71	56.25	100	97.92	51.52
			(9 SNPs)				(7 SNPs)				(7 SNPs)	
One rule	76.04	23.96	-	-	77.08	63.54	-	-	57.29	71.88	-	-
PART	55.21	54.17	96.88	57.69	56.25	62.5	88.54	60.71	67.71	53.13	93.75	66.67
			(9 SNPs)				(6 SNPs)				(9 SNPs)	
Random forest	46.88	96.88	93.75	**88.46**	63.54	85.42	88.54	**85.71**	54.17	94.79	90.63	**87.88**
			(14 SNPs)				(10 SNPs)				(15 SNPs)	
Random tree	45.83	67.71	94.79	53.85	54.17	69.79	94.79	57.14	59.38	66.67	95.83	48.48
			(6 SNPs)				(7 SNPs)				(7 SNPs)	
RIPPER	57.29	47.92	95.8	50	57.29	62.5	95.83	39.29	53.13	58.33	93.75	63.64
			(7 SNPs)				(8 SNPs)				(7 SNPs)	
SGD	54.17	100	97.92	65.38	63.54	100	98.96	57.14	57.29	100	98.96	63.64
			(8 SNPs)				(7 SNPs)				(8 SNPs)	
SMO	43.75	100	98.96	53.85	64.58	100	96.88	53.57	56.25	100	97.92	54.55
			(6 SNPs)				(5 SNPs)				(8 SNPs)	
Zero rule	52.08	52.08	-	-	64.58	64.58	-	-	56.25	56.25	-	-

Total SNP: 268,679 SNP.

GWAS, selection: Diabetes mellitus 2,848 SNPs, Cataract 2,878 SNPs, Hypertension 2,883 SNPs.

LIBSVM, A Library for Support Vector Machines; SGD, Stochastic gradient descent SVM; SMO, sequential minimal optimization logistic; KNN, K-nearest neighbors; LWL, locally weighted learning; RIPPER, repeated incremental pruning to produce error reduction; PART, Pruning rule-based classification tree.

Bold values indicate the highest accuracy within feature-selection method.

#### 3.4.1 Model comparison across feature-selection strategies

##### 3.4.1.1 Type 2 diabetes

**FIGURE 4:**
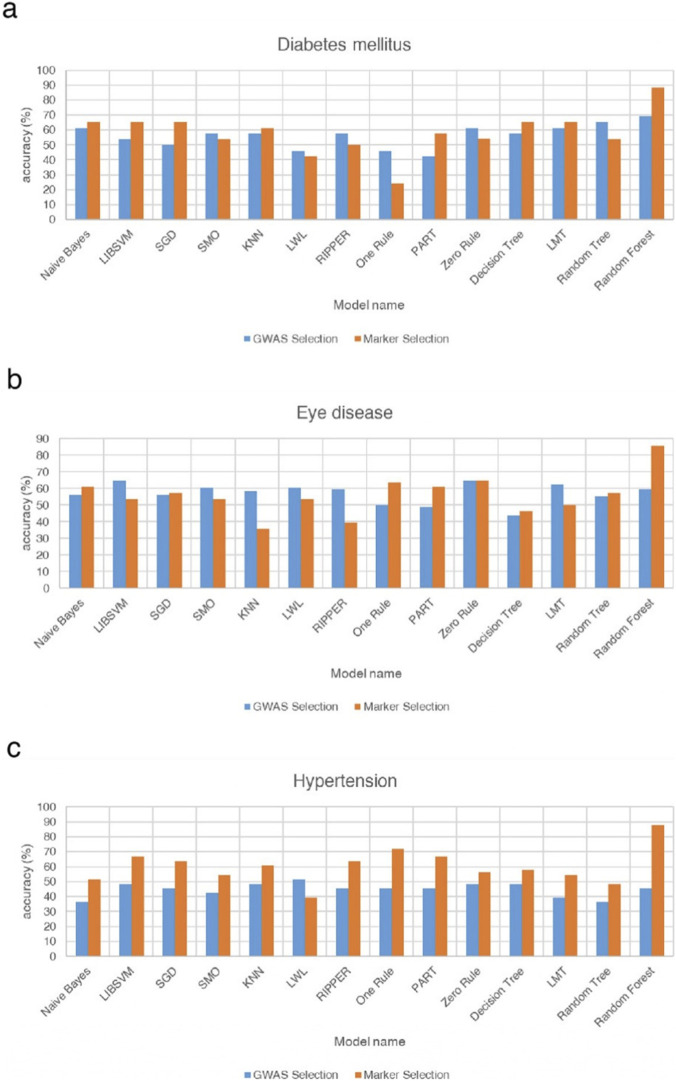
Comparison of the differences in accuracy rates among 14 models for type 2 diabetes, eye diseases, and hypertension under the filtering methods of genome-wide association analysis and machine learning. This figure compares the accuracy of three different disease models via GWAS selection and marker selection methods. **(a)** Shows the accuracy percentages of various models in predicting diabetes. Among these models, the Random Forest model achieves the highest accuracy, nearly 90%, when the marker selection method is used. **(b)** Presents the accuracies of the different models in predicting eye diseases. Similar to the diabetes models, the Random Forest model again has the highest accuracy, close to 85%, when the marker selection method is applied. **(c)** Illustrates the accuracy of various models in predicting hypertension. The Random Forest model again has the highest accuracy, approaching 90%, when the marker selection method is used. Compared with the diabetes and eye disease models, most models for hypertension prediction generally exhibit greater accuracy when the marker selection method is used than when the GWAS selection method is used. These findings suggest that the marker selection method may be more effective in predicting hypertension.

Accuracies across classifiers ranged from approximately 40%–90%, reflecting substantial heterogeneity across algorithms and feature sources (Figure 4a). Most models (e.g., Naïve Bayes, LIBSVM, SGD, KNN, PART, Decision Tree, LMT, Random Forest) achieved higher accuracy with marker-selected SNPs, whereas several others-including SMO, LWL, RIPPER, One Rule, and Random Tree-performed better with GWAS-selected features. This pattern indicates that algorithms differ in how they leverage redundant or interaction-enriched SNP signals. The Random Forest model achieved the highest and most stable performance-close to 90% with marker-selected SNPs and above 70% with GWAS-selected SNPs-highlighting its robustness and capacity to capture nonlinear, multi-locus interactions. In examining individual model behaviors, several distinct patterns were observed. PART and C4.5 decision trees showed clear improvement when trained with marker-selected SNPs, whereas LMT performed better with GWAS-selected features, consistent with its emphasis on additive signal structures. Linear and distance-based models-including LIBSVM, SGD, KNN, and Naïve Bayes-achieved higher accuracy with marker-selected SNPs, reflecting the advantage of reduced dimensionality and lower SNP-to-SNP correlation. In contrast, SMO displayed slightly better performance with GWAS-selected SNPs, suggesting closer alignment with the additive effects prioritized by traditional GWAS filtering. The Zero Rule classifier showed different accuracy levels between the two feature sets, a result driven by class-balance differences rather than SNP information. Several simpler or locality-dependent algorithms-such as Random Tree, RIPPER, LWL, and One Rule-performed better with GWAS-selected SNPs, likely because these methods rely on redundant or highly correlated variants that are removed during marker-based filtering.

##### 3.4.1.2 Eye diseases

Accuracies ranged from approximately 40%–85%, indicating considerable variability across classifiers (Figure 4b). Seven models performed better with marker-selected SNPs, six favored GWAS-selected SNPs, and one (Zero Rule) showed nearly identical results. The Random Forest model once again achieved the highest accuracy (greater than 80%) with marker-selected SNPs, while its GWAS-based performance remained among the top tier. Decision Tree, Random Tree, PART, Naïve Bayes, and SGD also improved under marker selection, suggesting that feature condensation and reduced redundancy enhanced decision purity and simplified probabilistic or gradient-based assumptions. In contrast, several models-including LIBSVM, SMO, LMT, KNN, LWL, and RIPPER-performed slightly better with GWAS-selected SNPs, implying reliance on the additive or locally redundant information preserved by traditional GWAS filtering. The One Rule classifier showed a notable improvement of more than ten percentage points under marker selection, indicating that even a single-feature rule can become predictive when the selected SNPs carry highly condensed information content. The Zero Rule baseline remained nearly identical between the two feature sets, confirming that performance differences were driven by algorithmic behavior rather than class imbalance. Overall, eye disease prediction appears to involve both additive and interaction-driven architectures, underscoring potential benefits from hybrid feature-selection frameworks that combine GWAS- and marker-based strategies.

##### 3.4.1.3 Hypertension

In contrast to the other diseases, hypertension exhibited a consistent, unidirectional trend - all models except LWL performed better with marker-selected SNPs (Figure 4c). Accuracies ranged from approximately 30%–90%, with the Random Forest model using marker-selected SNPs achieving the highest accuracy (about 90%), while Naïve Bayes and Random Tree under GWAS selection represented the lower bound (approximately 30%). Even the Zero Rule baseline showed improvement under marker selection, indicating enhanced separability within the refined feature space. The strongest gains were observed for Random Forest, PART, Decision Tree, RIPPER, Random Tree, KNN, and One Rule, confirming that ensemble, distance-based, and rule-based learners benefit most from the removal of noisy SNPs and the retention of interaction-enriched loci. Linear, kernel-based, and hybrid logistic-tree models (LIBSVM, SMO, SGD, and LMT) also improved markedly, suggesting greater stability of decision boundaries and kernel mappings after de-correlation. The sole exception, LWL, performed better under GWAS selection, likely due to its reliance on local redundancy that was attenuated by marker filtering. Collectively, these findings indicate that hypertension prediction is dominated by polygenic, weak-effect interactions, for which marker-based selection provides a consistent advantage.

#### 3.4.2 Validation and model robustness

To further assess model stability, the final Random Forest classifiers for the three diseases were re-evaluated using both leave-one-out cross-validation (LOOCV) and a 10-by-10 stratified cross-validation framework. External performance was assessed through 1,000 stratified bootstrap resampling iterations on the TWB Hakka cohort (n = 96), maintaining the original case–control ratio. The results (Table 2) demonstrated consistent accuracy and AUC across validation frameworks, confirming the robustness and reproducibility of the models. Despite the high point estimates of accuracy and AUC, the relatively wide confidence intervals reflect the uncertainty inherent in a small discovery cohort. Therefore, these results should be considered preliminary signals rather than definitive predictive performance.

**TABLE 2:** Performance of the final random forest models under internal and external evaluation.

Disease	Model	External evaluation (1,000× stratified bootstrap)			
		Accuracy		AUC	
		Mean ± SD	95% CI	Mean ± SD	95% CI
Type 2 diabetes	LOOCV	0.86 ± 0.09	0.67–0.97	0.94 ± 0.05	0.80–1.00
	10 × 10 CV	0.86 ± 0.09	0.68–0.97	0.94 ± 0.05	0.80–1.00
Eye diseases	LOOCV	0.85 ± 0.07	0.69–0.96	0.93 ± 0.04	0.83–0.99
	10 × 10 CV	0.83 ± 0.07	0.68–0.94	0.92 ± 0.04	0.83–0.99
Hypertension	LOOCV	0.89 ± 0.03	0.83–0.94	0.97 ± 0.01	0.94–1.00
	10 × 10 CV	0.90 ± 0.03	0.84–0.95	0.98 ± 0.01	0.95–1.00

The SNP, subsets for type 2 diabetes, eye disease, and hypertension were derived once using LOOCV-based wrapper feature selection on the discovery cohort and were fixed for all subsequent analyses. LOOCV, and 10 × 10 cross-validation reflect internal evaluation of the final models without re-running feature selection. External robustness was assessed using 1,000× stratified bootstrap resampling on the TWB, hakka cohort, using the same fixed SNP, subsets.

### 3.5 eQTL analysis reveals biological pathways associated with disease-related SNPs

To elucidate the potential functional roles of the SNPs identified in our analyses, we integrated eQTL data from the GTEx project (v10) to determine whether these variant SNPs influence gene expression in tissues relevant to the studied diseases. Among the 39 disease-associated SNPs, 13 were identified as cis-eQTLs for nearby genes (Supplementary Table S3).

For T2D, several SNPs exhibited eQTL effects in metabolically active tissues. Among these, the rs12121653 variant was associated with decreased expression of lysine demethylase 5B (KDM5B) in skin and esophageal tissues. KDM5B is a demethylase that targets Histone H3 Lysine 4 (H3K4). Experimental studies have shown that KDM5B-deficient mice display impaired insulin secretion; however, increased insulin sensitivity compensates for the maintenance of normoglycemia, even under a high-fat diet. These findings suggest that genetic variation may indirectly influence glucose homeostasis by modulating KDM5B expression, highlighting the interplay between epigenetic regulation and metabolic homeostasis (Backe et al., 2019). Additionally, rs12121653 showed a weaker cis-eQTL signal with the pseudogene MGAT4EP in testis tissue, although the biological relevance of this association remains unclear. Collectively, these results highlight KDM5B as the principal gene regulated by rs12121653, linking epigenetic modulation to glucose metabolism and energy balance in the Hakka cohort. For eye diseases, although the GTEx dataset lacks retinal tissue data, certain SNPs exhibited eQTL effects in blood and vascular tissues, suggesting a potential systemic influence on eye pathology. The absence of a mutation at rs6491129 (i.e., the wild-type genotype) in individuals with eye diseases was associated with upregulated expression of Cyclin-dependent kinase 8 (CDK8) in arterial and aortic tissues. CDK8 can promote Vascular Endothelial Growth Factor (VEGF) expression via the β-catenin-KLF2 axis (Wei et al., 2018; Menzl et al., 2019), thereby driving angiogenesis, which is implicated in retinal diseases such as diabetic retinopathy (DR) and age-related macular degeneration (AMD) through increased VEGF signaling (Callan et al., 2025). Additionally, rs6676790 was associated with the expression of LINC02772 in whole blood, potentially indicating an underlying immunoregulatory component in susceptibility to eye diseases. For hypertension, eQTL analysis revealed several SNPs affecting the expression of genes involved in vascular pathways. Among these variants, the rs6500596 variant was significantly associated with reduced expression of Heme oxygenase 2 (HMOX2) in arterial tissues, particularly in the aorta. HMOX2 belongs to the heme-degrading enzyme family and primarily catalyzes the breakdown of heme into biliverdin, free iron ions, and carbon monoxide (CO). CO exerts multiple cardiovascular protective effects, including promoting vascular smooth muscle relaxation, inhibiting vasoconstrictive responses, and reducing the generation of reactive oxygen species (ROS), thereby maintaining normal vascular tone and endothelial function. When HMOX2 expression is suppressed or its activity is reduced, CO production diminishes, leading to impaired vasodilation and compromised antioxidant defense. These pathological changes increase vascular tension and may ultimately increase the risk of hypertension (Muñoz-Sánchez and Chánez-Cárdenas, 2014).

Overall, these eQTL findings suggest that genetic variants associated with diabetes, eye diseases, and hypertension are linked to biological pathways involving epigenetic regulation, mitochondrial function, and vascular remodeling. These insights bridge the statistical associations identified through GWAS with potential mechanistic pathways underlying disease pathogenesis, underscoring the biological plausibility of the risk loci identified.

## 4 Discussion

This study revealed that, within the Taiwanese Hakka population, patients with T2D commonly exhibit a high degree of comorbidity with hypertension and eye diseases. This finding aligns with previous observations in Chinese populations, which documented systemic pathological mechanisms underlying such associations (Stram et al., 2018; Chang, 2010; Yin et al., 2020; Xu et al., 2024; Othman et al., 2025). In particular, chronic inflammation, endothelial dysfunction, and vascular injury induced by T2D may serve as shared pathophysiological bases for the increased risk of multiple diseases, highlighting the urgent need for integrated strategies to manage multisystem disorders. However, constructing disease prediction models based solely on significant SNPs identified through GWAS has clearly limited performance. Traditional GWAS-based approaches, while effective at identifying variants with strong marginal effects, fail to capture higher-order interactions or polygenic architectures, limiting their predictive utility. By contrast, our machine learning-based feature selection framework identified compact yet informative SNP subsets that substantially enhanced model robustness in both internal and external validation. The Random Forest model showed the most consistent relative performance across evaluation schemes, with cross-validation and bootstrap-estimated metrics indicating stable patterns rather than definitive predictive accuracy, given the limited sample size.

Notably, distinct patterns emerged across diseases: T2D and eye disease predictions reflected mixed additive and interaction-driven effects, whereas hypertension demonstrated a clear, unidirectional benefit from marker-based selection-suggesting its genetic risk is strongly polygenic and interaction-dependent. Through eQTL analysis, we linked rs12121653 to KDM5B and MGAT4EP, revealing potential mechanistic relevance to glucose metabolism and mitochondrial regulation. These results provide a functional context for statistical associations and support the biological plausibility of the selected markers. Integrating functional annotations with machine-learning-based feature selection thus offers a path toward biologically interpretable, population-specific risk models. Although full benchmarking against classical polygenic risk score (PRS) frameworks such as clumping and thresholding (C + T) or LDpred2 was not feasible due to the lack of large, ancestry-matched GWAS summary statistics, our exploratory analyses suggest that wrapper-based SNP selection could serve as a complementary alternative to conventional GWAS filtering strategies in small, ancestry-specific cohorts. Future studies utilizing large-scale East Asian summary data could extend this comparison and evaluate how PRS-based methods perform relative to model-dependent selection strategies in small, homogeneous populations. Despite these advances, several limitations remain. First, the discovery cohort consisted of only 96 Hakka individuals, which substantially limits statistical power and increases susceptibility to overfitting in machine-learning analyses. Although we applied LOOCV, fixed feature selection, and 1,000× bootstrap resampling to reduce this risk, the predictive performance should therefore be viewed as exploratory. Larger, independent Hakka datasets will be required to validate the stability of the selected SNP markers and model behavior. To ensure appropriate calibration of the GWAS findings, we incorporated sex as a covariate and evaluated alternative models with principal components. Appropriate calibration of the GWAS findings was confirmed through genomic control metrics, and the sex-adjusted model provided the most stable association statistics in this small and genetically homogeneous cohort. The sex-adjusted model therefore provided the most stable results, and the genomic control metrics (QQ plots and λGC values) indicated only minimal deviation from the null distribution. Additionally, the external TWB cohort exhibited class imbalance (approximately 10:1 controls to cases), which may have slightly inflated AUC estimates.

Future studies with larger, more balanced, and multi-ethnic samples will be essential to further assess generalizability. Methodologically, although the wrapper-based feature selection emphasized interpretability, it may not optimally preserve variance or control redundancy. Incorporating dimensionality reduction or multi-omics information may enhance both biological insight and predictive accuracy in future work.

## 5 Conclusion

In this study, we developed population-specific genetic risk prediction models for type 2 diabetes, hypertension, and eye diseases in the Taiwanese Hakka population by integrating genome-wide association analysis with machine-learning-based feature selection. This framework shows promise for improving prediction in small, ancestry-specific cohorts; however, the findings remain preliminary and require validation in larger and more diverse Hakka populations.

Across diseases, the models captured different underlying genetic architectures-interaction-driven effects were more prominent in hypertension, whereas mixed additive and epistatic components characterized T2D and eye diseases. Through eQTL annotation, key variants such as rs12121653-KDM5B and rs12121653-MGAT4EP were linked to functional pathways related to metabolic and mitochondrial regulation, providing biological insight into their potential roles in disease risk. Together, these findings highlight the utility of combining interpretable machine-learning strategies with biological annotation to uncover mechanistic and predictive insights in genetically homogeneous populations.

Although constrained by sample size and class imbalance, our results demonstrate that machine-learning-based SNP selection offers a scalable and generalizable approach for small, ancestry-specific cohorts. Future research should focus on validating these models in larger, multi-ethnic populations and integrating multi-omics data to refine predictive accuracy and clinical relevance. Ultimately, this framework represents a step toward population-tailored precision medicine and may serve as a foundation for developing clinically applicable genomic screening tools across diverse genetic backgrounds.

## Data Availability

The original contributions presented in the study are included in the article/Supplementary Material, further inquiries can be directed to the corresponding author.
