# Supplementary material for: Integrating GWAS and machine learning for disease risk prediction in the Taiwanese Hakka population
Source: Front Genet. 2025 Dec 4;16:1694084. doi: 10.3389/fgene.2025.1694084 (PMC12713315; doi:10.3389/fgene.2025.1694084)
Supplement: Supplementary file 1 [file Supplementaryfile1.docx]

Supplementary Material

**
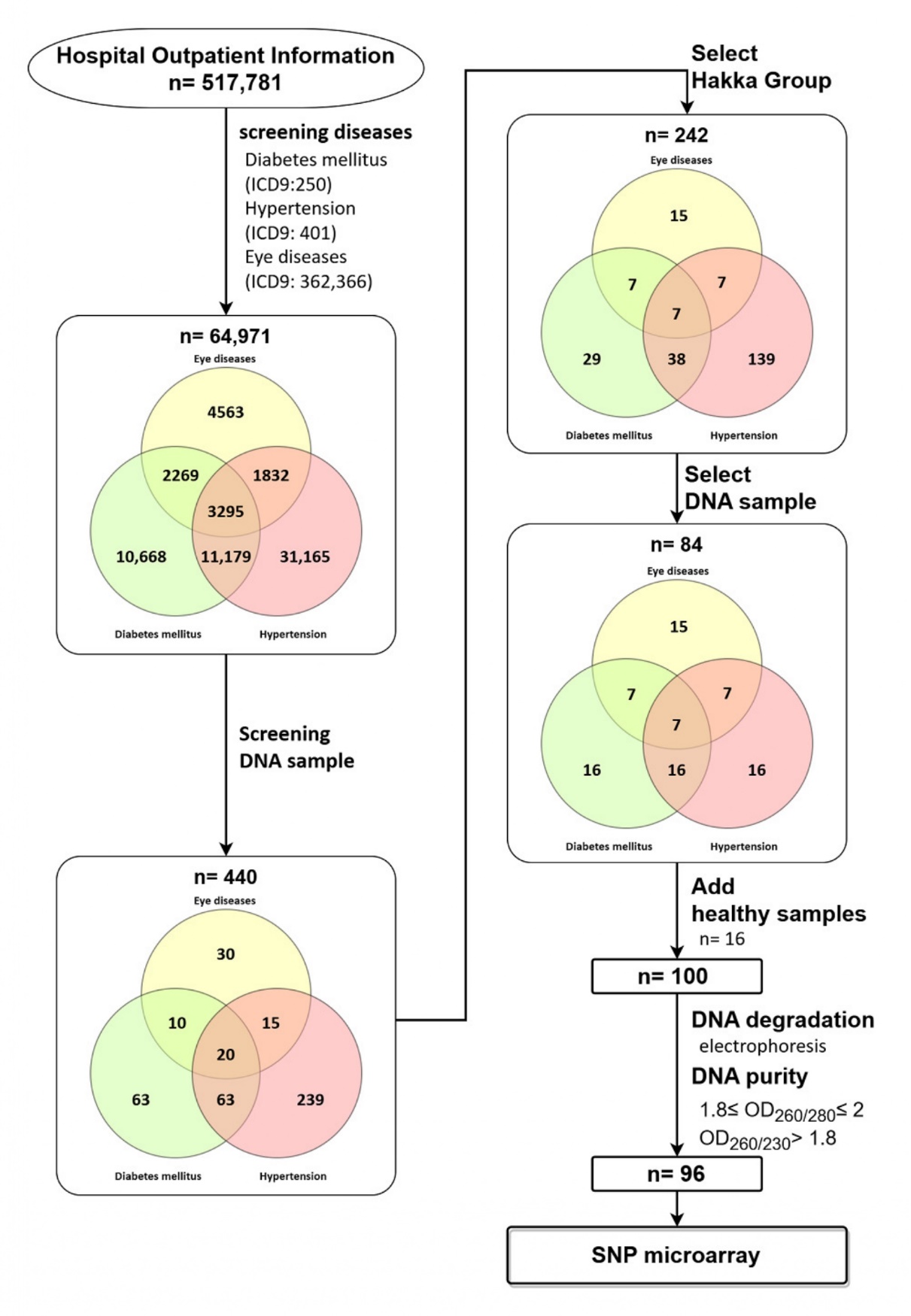
**

**Supplementary Figure S1. Flowchart for DNA sample selection and quality control (QC).** This flowchart illustrates the process of selecting DNA samples from hospital outpatient information for SNP microarray analysis. Initially, 64,971 patients with diabetes, hypertension, and eye diseases were selected from a pool of 517,781 outpatients. A total of 440 DNA samples were further chosen from these patients, including 242 samples from the Hakka ethnic group. These samples comprised patients with different combinations of diseases, ultimately resulting in to 84 DNA samples. To perform the SNP microarray analysis, 16 healthy samples were added. After DNA degradation and purity testing, a final selection of 96 samples was made for SNP microarray analysis.


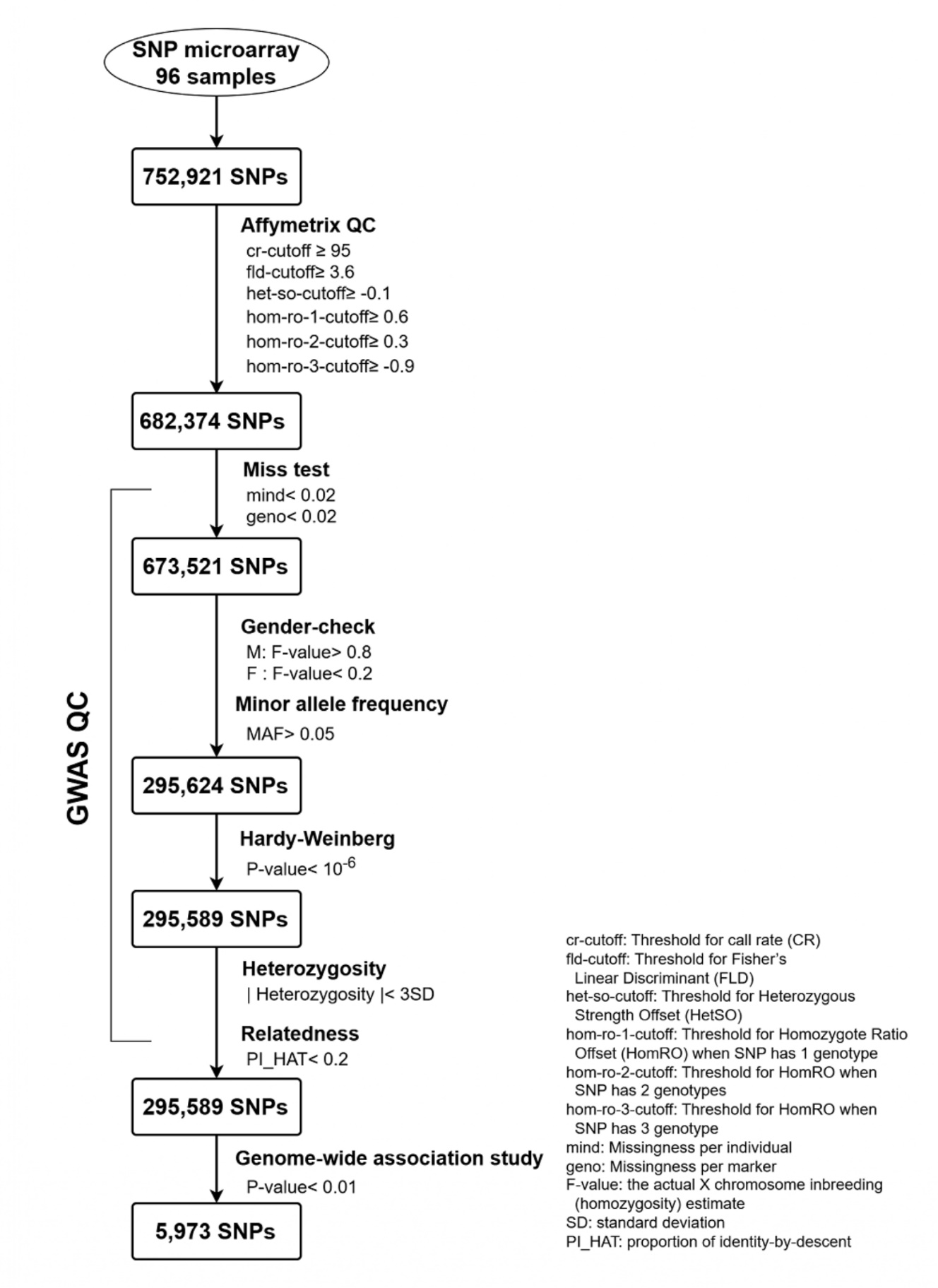


**Supplementary Figure S2. Flowchart for genome-wide association studies (GWAS).** This flowchart illustrates the quality control and screening process of 96 samples in SNP microarray analysis. Initially, 752,921 SNPs were obtained from the 96 samples. After Affymetrix quality control, 682,374 SNPs were retained. Next, a missingness test was conducted, which resulted in 673,521 SNPs. Next, sex checks and minor allele frequency filtering (MAF> 0.05) were performed, and 295,624 SNPs were retained. A Hardy‒Weinberg Subsequently, the Hardy-Weinberg equilibrium test was subsequently conducted, and SNPs with a P value less than 10-6 were retained, resulting in 295,589 SNPs were retained. After


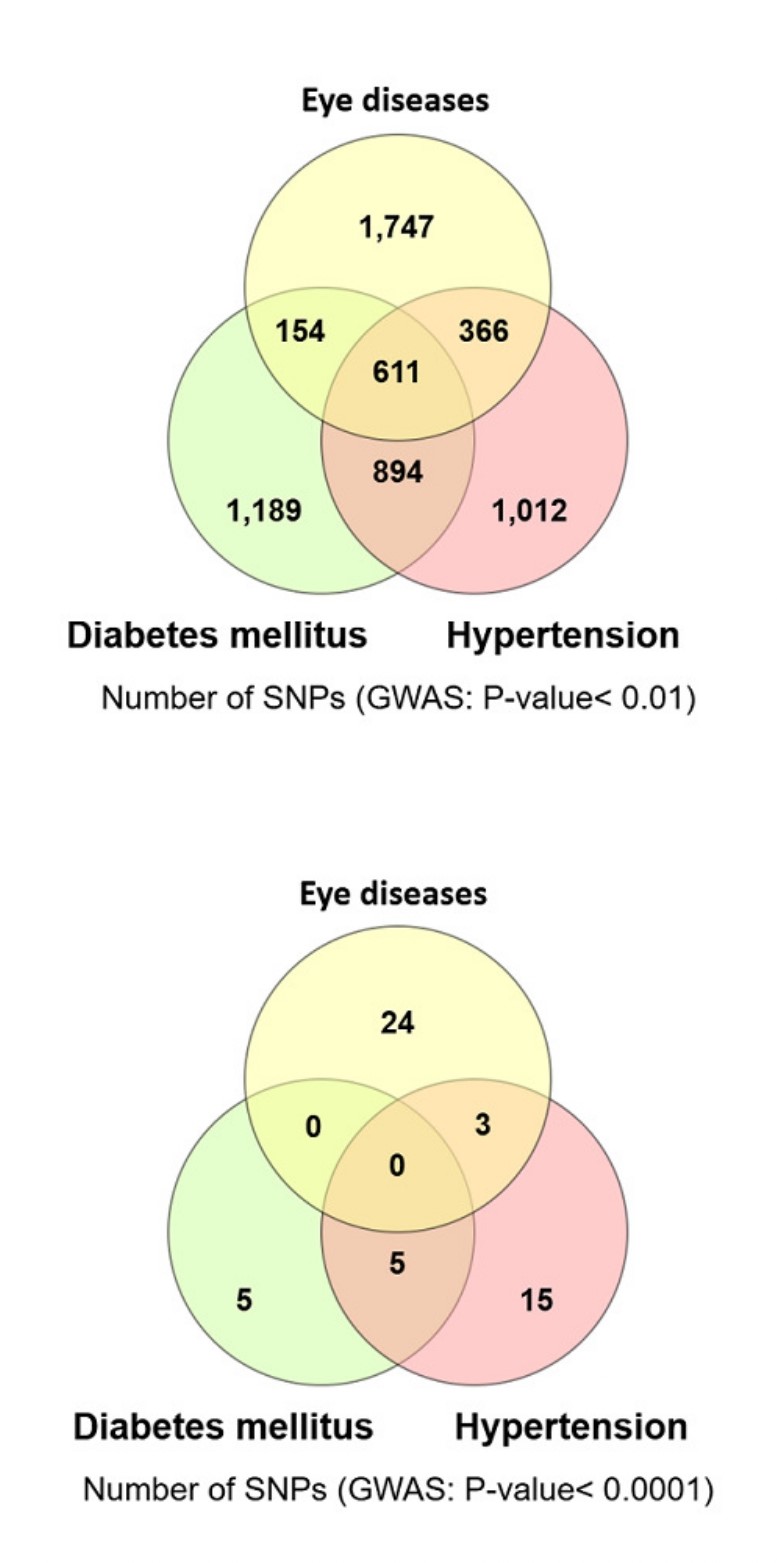


**Supplementary Figure S3. Overlap of SNPs identified under different significance thresholds.** This figure illustrates the number of SNPs associated with type 2 diabetes, eye diseases, and hypertension under different GWAS significance thresholds. Bar plots (or Venn diagrams, depending on the figure format) display SNP counts at P < 1 × 10⁻² (suggestive) and P < 1 × 10⁻⁴ (stringent), highlighting both shared and disease-specific variants. These results demonstrate how varying significance thresholds influence the identification of candidate SNPs across multiple diseases.


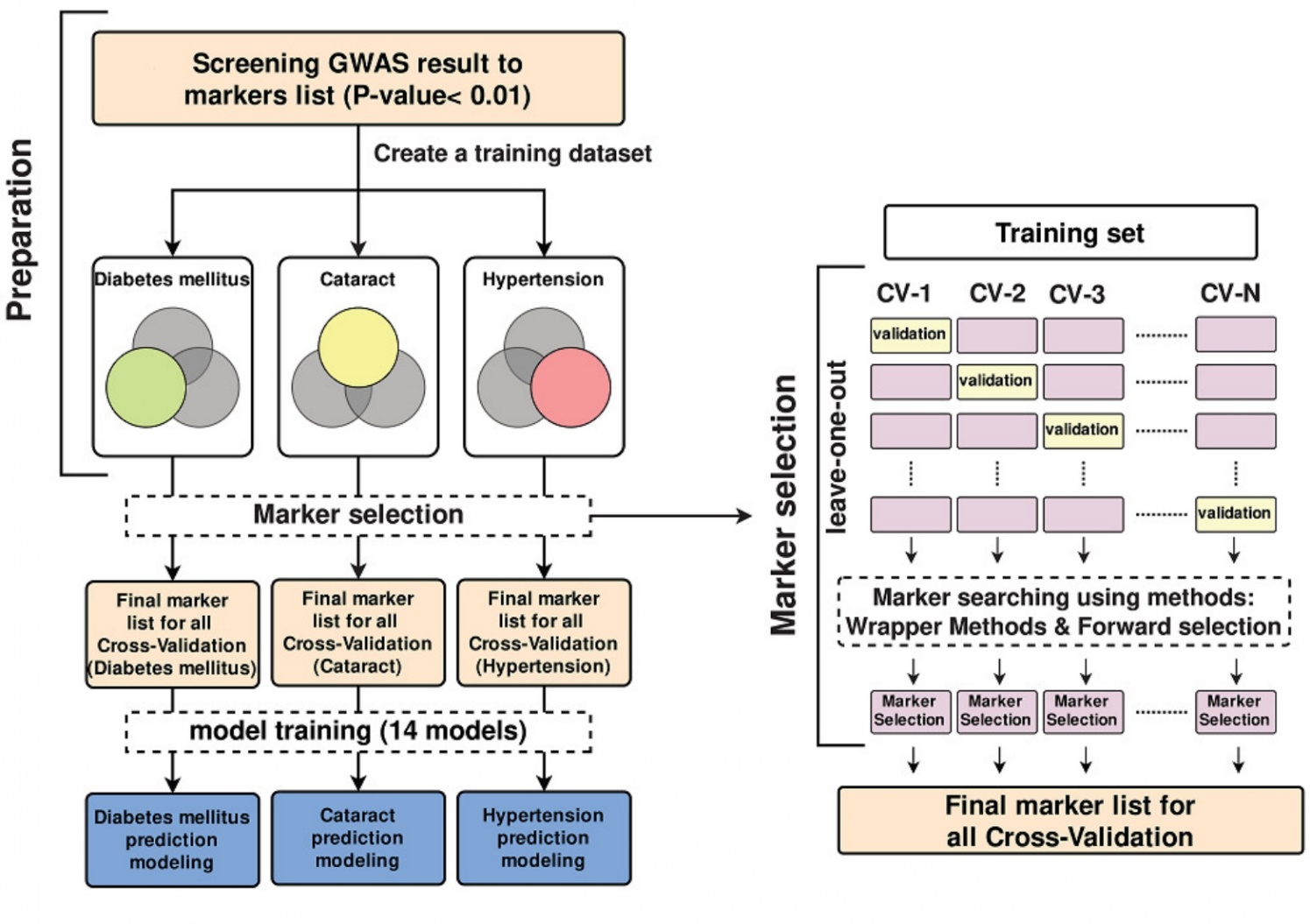


**Supplementary Figure S4. Flowchart of Model Training and the Feature Selection Process.** This figure illustrates the process of constructing disease prediction models for diabetes, cataracts, and hypertension. The entire workflow is divided into a preparation phase and a marker selection phase, culminating in model training. First, in the preparation phase, markers with P-values less than 0.01 were filtered from the GWAS results. These markers are used to create training datasets. Separate training datasets are established for each disease: diabetes, cataracts, and hypertension. Next, in the marker selection phase, the cross-validation (CV) method is employed for marker screening. Specifically, the training dataset is divided into multiple subsets (CV-1, CV-2, CV-3, ... CV-N), with each subset used to validate the selection of markers. Wrapper methods and forward selection are implemented to screen the markers, ultimately determining the final marker list for each disease. Finally, the training of the models is conducted via the finalized marker lists. For each disease (diabetes, cataracts, and hypertension), 14 different predictive models are trained to increase the accuracy and stability of the predictions.


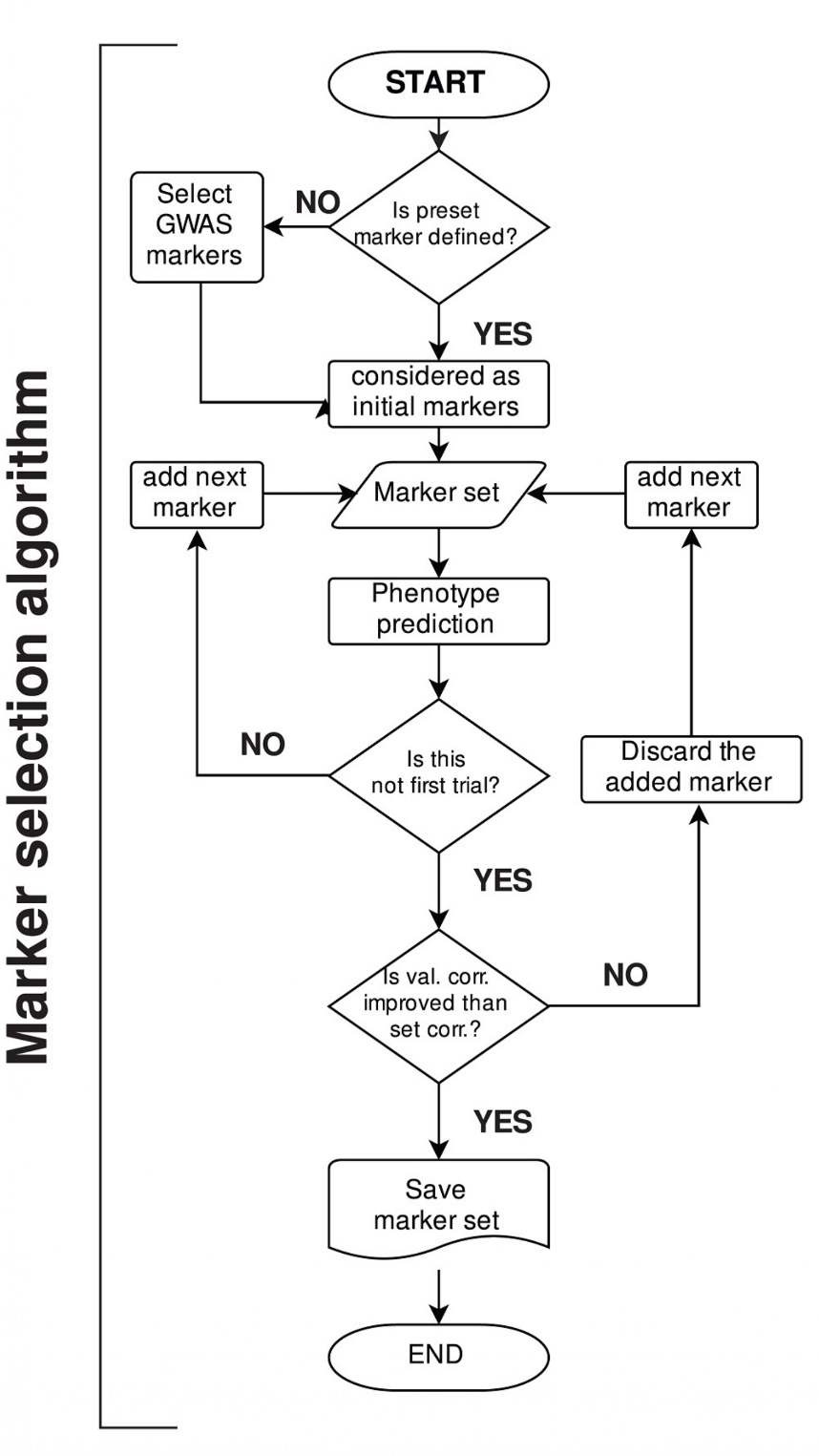


**Supplementary Figure S5. Logic diagram for machine learning model training and feature selection.** This flowchart illustrates the steps of a marker selection algorithm. The entire process begins with "Start" and proceeds through a series of decision points and operations to determine the most suitable set of markers. First, the algorithm checks whether predefined markers are already specified. If not, it selects GWAS markers and considers them as the initial markers. The process then moves into the marker set phase and proceeds with phenotype prediction. After phenotype prediction, the system checks if this is the first attempt. If it is not the first attempt, it compares whether the validation correlation has improved over the set correlation threshold. If there is an improvement, the marker set is saved, and the process ends. If there is no improvement, the newly added marker is discarded, and the next marker is added. This cycle continues until the optimal marker set is identified.

**Supplementary Table S1. Cross-validation accuracy of 14 machine learning models using GWAS-selected SNPs.** This table summarizes the predictive performance of 14 machine learning algorithms constructed with SNPs selected at a GWAS significance threshold of P < 1×10⁻⁴. Results are shown for type 2 diabetes, eye diseases, and hypertension, including internal cross-validation accuracy and external validation against Taiwan Biobank (TWB) data.

**Supplementary Table S2. SNP combinations selected by the Random Forest model.** This table lists the optimal SNP subsets identified by the Random Forest model for type 2 diabetes, eye diseases, and hypertension. For each SNP, genomic position, alleles, associated gene(s), GWAS P-values, GTEx eQTL status, and disease-specific intersections are provided. These markers represent compact yet predictive panels that improve model performance over GWAS-selected SNPs.

**Supplementary Table S3. Disease-associated SNPs with cis-eQTL effects identified in GTEx.** This table reports cis-eQTL relationships between disease-associated SNPs and nearby genes, as identified in GTEx v10. For each SNP–gene pair, genomic annotations (gene ID, position, biotype), effect size (beta, slope), statistical significance (p-values, q-values), and tissue-specific associations are shown. These results highlight potential functional mechanisms through which SNPs influence gene expression relevant to the studied diseases.

**Supplementary Table S4. Genomic inflation factors (λGC) under different covariate adjustment models for the three traits analyzed in this study.** This table summarizes the genomic inflation factors (λGC) obtained from four GWAS covariate adjustment strategies: (1) sex only, (2) sex + PC1-PC3, (3) sex + PC1-PC5, and (4) sex + PC1-PC10. λGC was computed separately for type 2 diabetes, eye diseases, and hypertension. Across all traits, the sex-only model exhibited λGC values close to 1.02-1.04, indicating minimal deviation from the null expectation. In contrast, incorporating increasing numbers of principal components resulted in progressively higher λGC values-reaching approximately 1.13-1.16 when PC1-PC10 were included-reflecting instability arising from over-correction in the small, genetically homogeneous discovery cohort. Based on these findings, the sex-adjusted model was selected as the primary GWAS framework due to its better calibration and interpretability in this setting.
